# Animal Models for the Study of Gaucher Disease

**DOI:** 10.3390/ijms242216035

**Published:** 2023-11-07

**Authors:** Or Cabasso, Aparna Kuppuramalingam, Lindsey Lelieveld, Martijn Van der Lienden, Rolf Boot, Johannes M. Aerts, Mia Horowitz

**Affiliations:** 1 Shmunis School of Biomedicine and Cancer Research, Faculty of Life Sciences, Tel Aviv University, Ramat Aviv 69978, Israel; orcaba@gmail.com (O.C.); aparnak@mail.tau.ac.il (A.K.); 2 Leiden Institute of Chemistry, Leiden University, 9502 Leiden, The Netherlands; l.t.lelieveld@lic.leidenuniv.nl (L.L.); m.j.c.van.der.lienden@lic.leidenuniv.nl (M.V.d.L.); r.g.boot@lic.leidenuniv.nl (R.B.)

**Keywords:** *GBA1*, glucocerebrosidase (GCase), glucosylceramide (GlcCer), misfolding, ER stress, unfolded protein response (UPR), inflammation, knockout animals, knockin animals

## Abstract

In Gaucher disease (GD), a relatively common sphingolipidosis, the mutant lysosomal enzyme acid β-glucocerebrosidase (GCase), encoded by the *GBA1* gene, fails to properly hydrolyze the sphingolipid glucosylceramide (GlcCer) in lysosomes, particularly of tissue macrophages. As a result, GlcCer accumulates, which, to a certain extent, is converted to its deacylated form, glucosylsphingosine (GlcSph), by lysosomal acid ceramidase. The inability of mutant GCase to degrade GlcSph further promotes its accumulation. The amount of mutant GCase in lysosomes depends on the amount of mutant ER enzyme that shuttles to them. In the case of many mutant GCase forms, the enzyme is largely misfolded in the ER. Only a fraction correctly folds and is subsequently trafficked to the lysosomes, while the rest of the misfolded mutant GCase protein undergoes ER-associated degradation (ERAD). The retention of misfolded mutant GCase in the ER induces ER stress, which evokes a stress response known as the unfolded protein response (UPR). GD is remarkably heterogeneous in clinical manifestation, including the variant without CNS involvement (type 1), and acute and subacute neuronopathic variants (types 2 and 3). The present review discusses animal models developed to study the molecular and cellular mechanisms underlying GD.

## 1. Introduction

Since its first report in 1882 by Ernest Gaucher, our understanding of the mechanisms underlying Gaucher disease (GD) has continuously grown. GD, inherited as an autosomal recessive disorder, is considered a common lysosomal storage disease (LSD) [1]. It results from mutations in *GBA1*, the gene encoding lysosomal acid β-glucocerebrosidase (GCase, EC 3.2.1.45 https://enzyme.expasy.org/EC/3.2.1.45, accessed on 3 April 2023), a 497 amino acid glycoprotein with four N-linked glycans, and a characteristic (α/β)8 TIM barrel catalytic domain. In the catalytic pocket, Glu340 acts as the catalytic nucleophile, and Glu235 serves as the acid/base residue for GCase [2,3,4,5]. The lysosomal activity of GCase depends on the presence of a small molecular weight activator protein, saposin C, which is a cleavage product of prosaposin, encoded by the prosaposin gene (PSAP) [6,7,8]. Prosaposin is a ~70 kDa highly conserved protein that is either transported to lysosomes or secreted from the trans-Golgi network. As a secreted protein, prosaposin is found in many secretory fluids, including cerebrospinal fluid, semen, milk, pancreatic juice, and bile [9]. Secreted prosaposin has been identified as a neurotrophic factor capable of promoting cell survival, neurite outgrowth, and differentiation in cholinergic cells [10] and protects neurons against oxidative stress [11]. In lysosomes, prosaposin is proteolytically processed to generate four cleavage products known as saposins A, B, C, and D. Each saposin is approximately 80 amino acids long with nearly identical placement of cysteine residues and glycosylation sites, which serve as an activator of another lysosomal hydrolase [12]. Saposin A enhances β-galactocerebrosidase (deficient in Krabbe disease) [13], saposin B activates arylsulfatase A (mutated in metachromatic leukodystrophy) [14,15,16], saposin C activates GCase [12,17], and saposin D activates ceramidase (mutated in Farber disease) [18]. A deficiency in saposin C leads to the development of GD. In most cases, it is a neuronopathic form of the disease [19,20,21]; however, two siblings displayed type 1 GD due to two mutations in the saposin C domain of prosaposin [22].

As a lysosomal enzyme, GCase is synthesized on the endoplasmic reticulum (ER)-bound polyribosomes. When normal GCase is correctly folded in the ER, it is transported to lysosomes, where it hydrolyzes glucosylceramide (GlcCer). The transport of GCase from the ER to lysosomes in fibroblasts depends on the LIMP2 membrane protein [23], encoded by *SCARB2*. Interestingly, the presence of biallelic mutations in the *SCARB2* gene leads to action myoclonus-renal failure (AMRF) [24]. These patients present with low GCase activity in fibroblasts (less than 10% of controls) and 30% of normal GCase activity in macrophages [25]. In LIMP2 KO mice, increased levels of GlcCer were found in the liver and lungs, whereas no accumulation was detected in the spleen, kidney, or brain [23]. These results may indicate the existence of yet another unknown GCase receptor in macrophages. 

**Figure 1:**
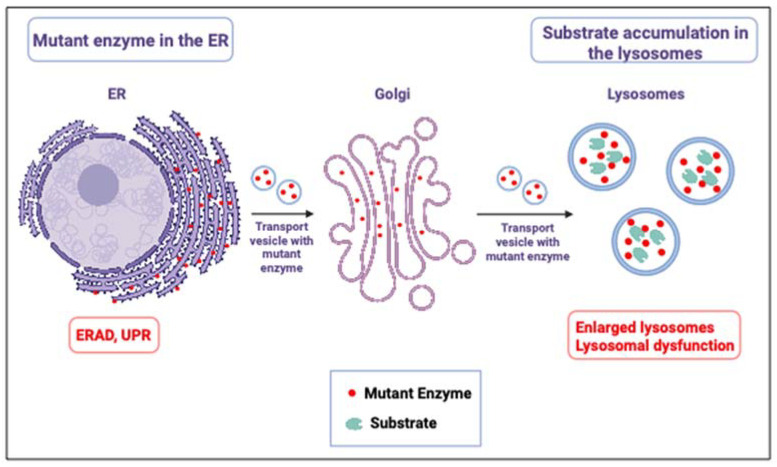
Two pathologies in GD. In GD, there are two pathologies: A. ER retention of misfolded GCase molecules in the ER, leading to ER stress and ER stress response, known as the unfolded protein response (UPR) [36,37], and B. Due to the decreased number of GCase molecules in the lysosomes and their decreased ability to normally degrade the substrate, there is substrate accumulation.Illustration was produced using BioRender.

Mutant GCase molecules, recognized as misfolded in the ER, are retained there for several folding attempts. Retention of mutant misfolded GCase in the ER causes stress and activates the UPR machinery [26,27], part of which is ER-associated degradation (ERAD) [28]. Due to ERAD, a decreased amount of mutant GCase is trafficked to the lysosomes, which leads to lysosomal accumulation of GlcCer particularly in tissue macrophages. The characteristic lipid-laden macrophages of Gaucher patients are called Gaucher cells [29] (Figure 1). Deacylation of GlcCer to glucosylsphingosine (GlcSph) by lysosomal acid ceramidase leads to the accumulation of GlcSph mainly in the kidney, liver, and spleen and to its secretion from cells [30].

A large number of mutations are known to date in *GBA1:* 300 mutations have been published [31], and over 750 mutations are listed in the gnomAD browser (address: Genome Aggregation Database). The mutations cause extremely varied symptoms. GD is generally classified into three different clinical variants based on the absence (type 1 GD—GD1) or presence (type 2—GD2 and type 3—GD3) of neurological manifestations [32]. GD1 patients do not have any neurological symptoms, although they are predisposed to development of Parkinson disease [33]. GD2 results in premature death in the first few years of life [34], while GD3 patients develop a neurological disease at later ages, with a longer life expectancy than GD2 patients [35]. 

Type 1 GD was the first lysosomal storage disease for which macrophage-targeted enzyme replacement therapy was developed and applied with tremendous clinical success and outcome. Subsequently, substrate reduction therapy for this disorder became available. Chaperone-mediated therapy and gene therapy are in clinical trials.

The present review discusses published animal models that have been developed to study the molecular mechanisms underlying GD and to test different therapeutic modalities. 

## 2. Mouse Models for Gaucher Disease

### 2.1. Chemically Induced Mice Models

The first effort to generate GD animal model involved the inhibition of GCase activity with conduritol-B-epoxide (CBE), a non-competitive inhibitor of GCase [38]. In 1975, Kanfer et al. reported that daily injection of 100 mg/kg body weight CBE to 90-days-old C57/Rl mice for three weeks, resulted in up to 93% reduction in GCase activity with a two- to five-fold increase in GlcCer levels in the spleen, liver, and brain [39]. In 1978, Stephens et al. noted that the brain, liver, and spleen have different sensitivities to CBE, with the brain being the most sensitive [40]. 

Marshall et al. used CBE and GlcCer containing liposome-treated mice to study the applicability of gene therapy for GD. When adenoviral vector encoding human GCase was intravenously injected into these mice, higher amounts of GCase were detected in various affected tissues, which directed GlcCer clearance from the liver of these mice [41]. 

To understand the mechanism of neuronal cell death, CBE-treated mice were used to study the role of necroptosis in nGD brains [42]. The authors concluded that neuronal cell death occurs via the receptor-interacting protein kinase-3 (Ripk3) necroptotic pathway. Upon modulation of this pathway, the nGD mice had improved survival and motor coordination. The same group later documented that CBE-treated mice developed neuroinflammation, which culminated in the death of neuronal cells, recapitulating symptoms that occur in nGD [43]. 

Klein et al. studied the effect of CBE on the development of GD in 15 different inbred mouse strains injected with 25 mg CBE/kg body weight from post-natal day 8. The various strains exhibited different lifespans and there was no direct correlation between the levels of accumulated substrate and lifespan [44]. 

As mentioned, the hallmark of GD is the accumulation of the lipid-laden Gaucher cells in tissues. Gaucher cells are viable and secrete specific proteins into the circulation, chitotriosidase, and CCL18, being the most prominent ones that are presently used as GD biomarkers [45]. Unfortunately, chitotriosidase and CCL18 are not produced by lipid- laden macrophages in GD mice. A proteomics study led to the discovery of markedly increased glycoprotein non-metastatic B (GPNMB) in GD spleen, accompanied by elevated levels of a soluble fragment of GPNMB (s-GPNMB) [46]. Interestingly, mouse Gaucher cells also overproduce GPNMB and secrete s-GPNMB. Microglia may also produce GPNMB [47]. Zigdon et al. documented a correlation between nGD severity and GPNMB levels [48]. CSF GPNMB levels were higher in GD2 and GD3 patients than in age-matched controls. Moloney et al. showed that daily intraperitoneal injection of mice aged 2–3 months, for 28 days, with 100 mg/kg CBE led to a decrease in GCase activity and elevation in GPNMB levels in the brain, along with glial activation in several brain regions [49]. 

It is worth mentioning that CBE-treated cells or animals do not develop UPR since the inhibited GCase is not misfolded and is normally trafficked to the lysosomes. Thus, in cultured hippocampal neurons treated with 200 mM CBE for 4–9 days, UPR could not be recapitulated [50].

### 2.2. Chimeric Mice Models

In 2002, a non-genetic GD mouse model was developed by Beutler et al., who infused hematopoietic stem cells from fetuses, homozygous for *Gba1* KO, into irradiated mice, resulting in a chimeric mouse model [51]. These mice presented a deficiency in GCase activity in peripheral blood cells and in the spleen, accompanied by GlcCer accumulation, and they did not present any brain involvement, mimicking GD1.

### 2.3. Knockout (KO) and Conditional Knockout GD Models

In KO models, the activity of GCase is completely ablated owing to disruption of the mouse *Gba1* gene. In conditional KO models, the *Gba1* ortholog is destroyed at a controlled, specific time point and/or in specific tissues. It is worth mentioning that KO of human *GBA1* is incompatible with post-natal life. 

#### 2.3.1. KO Mice Models

The first knockout (KO) GD mouse model, produced through targeted disruption of the *Gba1* gene using homologous recombination, was developed in 1992. This KO mouse had <4% GCase activity compared to WT mice, and GlcCer accumulated mainly in the liver, brain, and lungs. It had a severe GD2 phenotype and died within 48 h due to an abnormal skin permeability caused by increased trans-epidermal water loss [52]. The animals mimicked the collodion baby, an extreme form of GD2 [53]. 

Orvisky et al. noted that KO mice, homozygous for a null allele, had approximately a 100-fold elevation of GlcSph in the brain, as well as elevated levels in other tissues. This accumulation was detected in utero by embryonic day 13 and increased progressively throughout gestation [54]. 

Hong et al. documented elevated levels of the proinflammatory cytokines IL-1α, IL-1β, IL-6, and tumor necrosis factor (TNF)-α in the fetal brains of KO GD mice, as well as elevated levels of secreted nitric oxide and reactive oxygen species [55]. In parallel, the same group documented that the expression of brain-derived neurotrophic factor (BDNF) and nerve growth factor (NGF) was reduced in the KO mice along with downregulation of ERK1,2 in their neurons [56]. 

Another study of *Gba1* KO mice showed that GlcCer-dependent inflammation was due to the activation of complement C5a and its receptor (C5aR1). When *Gba1* and *C5AR1* double KO mice were tested, reversal of GlcCer storage, inflammation, and proinflammatory cytokines was noted, and the mice lived longer, strongly suggesting that C5aR1 plays an important role in the activation of inflammation [57]. 

#### 2.3.2. Conditional KO Mice Models

Since the short lifespan was a major limitation of *Gba1* KO mice, conditional KO mice were generated. Enquist et al. generated the first *Gba1* conditional KO mouse by conditional deletion of exons 9–11 of the mouse Gba1 gene. The deletion was initiated after birth using the myxovirus resistance protein 1 (Mx1) promoter coupled to the Cre recombinase. To turn the Mx1 promoter on, in order to enable the deletion of exons 9–11 through Cre recombinase, a series of five polyinosinic–polycytidylic acid injections, starting within the first week of life, were used. This procedure maintained normal GCase activity during development and avoided disturbance of the skin barrier formation. These mice presented with GCase deficiency in the hematopoietic organs, massive infiltration of Gaucher cells in the bone marrow, liver, and spleen, and a significant increase in GlcCer. In addition to splenomegaly, the mice exhibited microcytic anemia mimicking GD1. Both, transplantation of WT bone marrow (BM) and gene therapy through retroviral BM transduction, corrected the GD phenotype. The gene therapy approach generated considerably higher GCase activity than the transplantation of WT bone marrow. Both therapeutic modalities normalized GlcCer levels with no infiltration of Gaucher cells in the BM, spleen, and liver, demonstrating correction at 5–6 months after treatment [58]. Similarly, upon lentiviral transduction of human *GBA1* into this mouse, clearance of GlcCer from BM, liver, and spleen was reported, which resulted in the reversal of splenomegaly, reduced Gaucher cell infiltration, and restoration of hematological parameters [47].

Relatively common among GD patients are multiple myeloma and B-cell lymphoma. Using the conditional KO generated by Enquist et al. to eliminate the expression of *Gba1* from the hematopoietic organs [58], Pavlova et al. observed that substrate reduction therapy with eliglustat tartrate (GENZ 112638), to inhibit GlcCer biosynthesis, suppressed the development of B-cell lymphoma and myeloma [59,60]. 

Sinclair et al. established hematopoietic and endothelial cell-specific conditional *Gba1* knockout models by conditional deletion of exons 9–11 in the *Gba1* gene, using the tyrosine kinase Tek (Tie2) or the monocyte-specific M lysozyme (LysM) promoters, respectively. To this end, loxP sites were inserted in intron 8 and after exon 11 of the *Gba1* gene. The loxP site after exon 11 was coupled to a neo gene flanked by Flp sites. After establishment of ES cells, the neo gene was excised by Flippase. Mice were generated and crossed with transgenic animals expressing Cre recombinase coupled to either the Tie2 or LysM promoters. These mice presented with GlcCer storage which led to progressive splenomegaly with Gaucher cell infiltration and moderate GlcCer storage in the liver by 26 weeks of age, with no significant bone marrow pathology [61]. Enquist et al. generated the first viable genetic nGD conditional mouse model in which a loxP-neo-loxP (lnl) cassette was inserted into the 8th intron of murine *Gba1* gene. This led to an abnormal splicing of the *Gba1* mRNA in all body tissues. To restore normal epidermal function, while maintaining its deficiency in all other tissues, these lnl mice were bred with a transgenic mouse, in which Cre recombinase expression was driven by K14 promoter, ensuring excision of the loxP-neo-loxP cassette and normal expression of *Gba1* in the skin. These mice (K14Lnl/lnl) exhibited rapid motor dysfunction associated with severe neurodegeneration, neuronal loss, and massive microglial activation and proliferation. These mice were used to test several therapeutic modalities. Thus, intracerebroventricular (ICV) administration of recombinant human GCase into these mice alleviated neuropathology in multiple brain regions [62]. Systemic administration of a novel GlcCer synthase inhibitor, GZ161, the first compound to cross the blood-brain barrier [63], resulted in reduction in GlcCer and GlcSph as well as CNS pathology, together with a significant increase in lifespan in these mice.

A second mouse model was established by crossing the lnl mice with nestin-Cre expressing animals (*Gba^flox/flox^*; nestin-Cre mice), recovering expression of *Gba1* in neuronal and macroglial cells, rendering GCase deficiency in microglial cells. These mice developed similar pathology as the first mouse model, but with delayed onset and slower disease progression. The authors indicated that the results suggested that GCase deficiency within microglial cells is not the primary determinant of the central nervous system pathology [64]. Further analysis of the Gba^flox/flox^; nestin-Cre mice revealed microglial activation and astrogliosis, which were correlated with neuronal loss [65]. In a later publication, using the same animal model, it was suggested that GlcCer storage in neurons, triggers a signaling cascade that activates microglia, which in turn releases inflammatory response, contributing to neuronal death [66]. 

Mistry et al. developed a conditional KO model using Mx1 promoter and deleted exons 8–11 of mice *Gba1* by administering polyinosinic–polycytidylic acid from post-natal day 2. This resulted in greater than 95% reduction in GCase activity in hematopoietic and mesenchymal cell lineages. GlcCer and GlcSph accumulated, inflammatory markers increased, and severe osteoporosis was observed. Interestingly, not only macrophages but also thymic T cells, dendritic cells, and osteoblasts were affected, which provided evidence for the involvement of multiple cell lineages [67].

Besides the *GBA1*-encoded lysosomal GCase, there is a distinct non-lysosomal glucosylceramidase, named *GBA2*, that also converts GlcCer to ceramide [68,69,70]. *GBA2*, a GH116 glycosidase with a catalytic (α/α)6 TIM barrel domain, is a very well conserved enzyme (from nematode to man). It is synthesized as a cytosolic protein and rapidly inserts into membranes with its catalytic pocket facing the cytosol. The enzyme has potent transglycosidase activity and can transfer glucose from GlcCer to cholesterol, thus generating glucosylated cholesterol (GlcChol) [71]. The role of *Gba2* during GCase deficiency has been investigated in GD mice. Mistry et al. reported that in mice deficient for *Gba2* and lacking *Gba1* in their hematopoietic stem cell lineage (Mx1-Cre; *Gba2^−/−^*), there were ameliorated bone abnormalities along with a complete reversal of hepatosplenomegaly, cytopenia, and partial reversal of hypercytokinemia [72].

A GD3 conditional KO mice model was generated by Pewzner et al. A cassette, containing a neo gene flanked by loxP sites, a tetracycline expressing gene, and a *Gba1* gene, was introduced into the ROSA26 locus. By crossing these animals with animals expressing Cre under the pGK promoter, the neo gene was deleted, which allowed for transcription/translation of the transactivator (tTA) (driven by the intact ROSA26 promoter), which promoted transcription and translation of GCase (*Gba^tg^* mice). Upon administration of doxycycline, it bound to the tTA, which reduced GCase expression. These mice were then used to generate *Gba^−/−^*, *Gba^tg^* mice. Upon administration of doxycycline, their GCase activity was ~30% and ~50% in brain and liver, respectively. In addition to neuronal deficits, bone deformities and dental manifestations were also observed [73]. 

Crosses between different GD mouse models yielded a range of animals with different phenotypes. *Gba1* rescue in microglia or neuron of *Gba^lnl/lnl^* mice by using Cx3cr1-Cre or nestin-Cre, respectively, prolonged survival, which was further enhanced upon treatment with brain-penetrant inhibitor of glucosylceramide synthase. It also reduced GlcCer and GlcSph accumulation, accompanied by reduced neuroinflammation and reduced serum neurofilament light chain (Nf-L) [74]. 

### 2.4. Knockin (KI) Mice Models

KI models, in which a known GD mutation is introduced into the animal *Gba1* ortholog, allow for better recapitulation of the condition in patients who, in most cases, have point mutations in their *GBA1* gene and show residual enzyme activity.

The first KI model was generated by Liu et al. [75], who introduced two known human nGD-causing mutations, L444P [76] and recNcil [77], into the mouse *Gba1* gene. Mice homozygous for the recNcil mutation showed 4–9% residual GCase activity [75] and GlcCer accumulation in the brain, liver, and skin. L444P homozygous mice had 20% of normal GCase activity and no detectable accumulation of GlcCer in the brain or liver. Unexpectedly, mice homozygous for either mutation died within 48 h of birth due to increased trans-epidermal water loss caused by defective GlcCer metabolism in the epidermis. The skin barrier abnormality was eliminated by breeding L444P heterozygous mice with mice carrying a GlcCer synthase-null gene (Ugcg) [78]. Offspring homozygous for the L444P mutation and heterozygous for the null Ugcg mutation reached adulthood with less GlcCer. The Ugcg KO gene was replaced with its normal counterpart to create L444P homozygous animals, some of which had long-term survival. The animals displayed systemic inflammation, including evidence of B-cell hyperproliferation with no significant accumulation of GlcCer in tissues or evidence of Gaucher cells. These results indicated that GCase deficiency, even in the absence of large amounts of sphingolipid storage, can trigger an inflammatory reaction.

Subsequently, mouse models with the point mutations: N370S, V394L, D409H, and D409V were established [79]. The N370S homozygous animals did not survive beyond the neonatal period, due to a skin abnormality. In the other homozygous mice, a few storage cells appeared in the spleen at ~7 months (D409H or D409V homozygotes) or at ~1 year (V394L homozygotes). D409V homozygous mice exhibited progressive accumulation of proteinase K-resistant α-synuclein/ubiquitin aggregates in hippocampal neurons, accumulation of GlcSph, and memory deficits [80].

The D409V KI mouse was crossed with the *Gba1* KO to create a D409V/null mouse. The development of a neuronopathic disease was evident in this mouse model, which presented a progressive accumulation of GlcCer and GlcSph in the brain [81]. The D409V/null model revealed altered expression patterns for approximately 0.9–3% of genes, mainly representing macrophage activation and immune response genes [82]. Both imiglucerase (Sanofi/Genzyme) and velaglucerase alpha (Shire) were effective in reducing GlcCer storage in the liver, spleen, and lungs of 3-month-old D409V/null mice. SRT, using eliglustat to inhibit GlcCer synthase [83], was also effective, albeit to a lesser degree than ERT [84]. Another mouse model, established by Sun et al. comprised the *Gba1* V394L homozygous mutation and a homozygous saposin C mutation, designated 4 L;C (V394L/V394L/saposin C^−/−^) [85]. The mice were viable and developed nGD with GlcCer and GlcSph accumulation, and progressive neurological deficits, including gliosis and paresis (weakened or impaired muscle movement). Treatment with ibiglustat, an SRT compound that crosses the blood-brain barrier [86], alleviated neurological signs with a marked reduction in the amounts of accumulating substrates. Treatment of the animals with isofagomine, a pharmacological chaperone that enhances GCase function [87,88,89,90] led to an increase in GCase activity and in lifespan of the animals, but the cerebral cortical GlcCer and GlcSph levels remained the same [91]. 

To conclude, the different GD mouse models revealed sites of substrate accumulation, development of osteoporosis, (neuro)inflammation and immune cell activation in GD, as well as the feasibility of new therapeutic modalities, especially gene therapy. 

## 3. Fish Models 

Fish, particularly zebrafish (*Danio rerio*), have become a popular vertebrate model for gaining insight into human diseases [92]. Zebrafish are teleost that naturally occur in South Asia and survive in different environments at temperatures ranging from 24 to 38 °C. Laboratory zebrafish are generally maintained at a fixed temperature of 28 °C, with controlled circadian rhythms, optimized water quality, and feeding [93]. Zebrafish can live up to 3–4 years, showing signs of aging at approximately 2 years [94]. 

The attractive features of zebrafish include cost-effective maintenance, fertility, ex vivo fertilization, and rapid development. Additional advantages of embryonic and larval zebrafish include *ex-uteral* development, transparency, small size, and ease of genetic manipulation. The genome of zebrafish has been elucidated with 25 chromosomes, and more than 26,000 protein-coding genes have been annotated. The conservation of genes between humans and zebrafish is high; 70% of human genes have at least one ortholog in the zebrafish genome. This seems even higher (approximately 82%) for genes implicated in human disorders [95]. Fish contain several organs and tissues analogous to those of mammals, including the brain, heart, liver, kidney, pancreas, intestinal tract, and spleen. Missing are the lung, skin with a stratum corneum, and the mammary gland. On the other hand, the zebrafish liver, similar to its human counterpart, contains hepatocytes, endothelial cells, and bile duct epithelial cells, but Kupffer cells are absent [96]. 

Several standard histopathological, biochemical, genetic, and analytical techniques can be used to study zebrafish. Numerous zebrafish lines have been generated, expressing fluorescent reporters that either mark specific cell types and subcellular compartments, or are under the regulation of an inducible promoter [97,98]. The generation of KO in zebrafish using CRISPR/Cas9 or overexpression of a given gene is straightforward and can be achieved in a matter of months.

**Figure 2:**
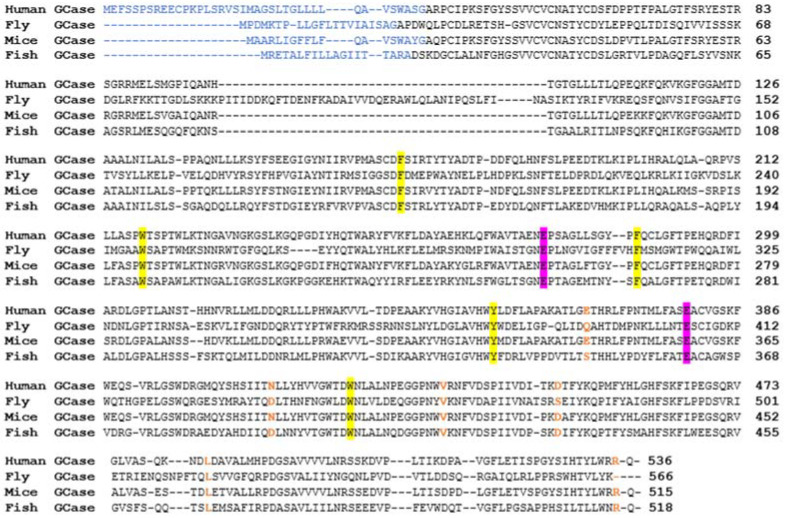
Multiple sequence alignment of active GCases between different organisms. MSA analysis of human, fly, mouse, and fish GCase sequences. The known human and predicted animal leader sequences are highlighted in blue. The two amino acids in the active site are highlighted in purple and the amino acids involved in stabilizing it are highlighted in yellow. The prevalent GD mutations E326, N370, V394, D409, L444, and R496 are shown in orange.

Zebrafish have one *GBA1* ortholog, *gba1*, with 10 exons on chromosome 16. It encodes a 518 amino acid protein with approximately 57% similarity to human GCase. The active site, along with the amino acids that stabilize it, is conserved among humans and zebrafish. The catalytic sites in fish and human GCases are conserved [E235 and E340 in humans [2]; E237 and E342 in zebrafish], as well as the amino acids that stabilize the active site (F128, W179, F246, Y313, W381 in human GCase and F130, W181, F248, Y315, W383 in fish GCase) (Figure 2).

Among the several residues involved in GD mutations, D409, R496, and L444 are conserved, whereas N370 is not. Aspartic acid (D370) is present instead of asparagine. Interestingly, N370 is not conserved in *Drosophila melanogaster,* as well. 

### 3.1. Pharmacological Zebrafish Models of Gaucher Disease

As described previously, CBE-induced GD models have been used for a long period but one of their major drawbacks is the off-target inhibition of other glucosidases. To specifically inactivate glycosidases of interest in zebrafish, small compound inhibitors (reversible or irreversible ones) have been designed, like cyclophellitol analogs. Artola et al. showed that the zebrafish GCase could be selectively inactivated by exposure of the animals to ME656, a cyclophellitol derivative and this led to a rapid increase in GlcCer and GlcSph accumulation [99]. The ME656 compound is derived from a fluorescent activity-based probe that was shown to selectively label GCase by covalent linkage to the catalytic nucleophile E366 [100,101].

### 3.2. Genetic Models of Gaucher Disease

Genetic diseases, including lysosomal disorders, can be generated in zebrafish using an injection of antisense morpholino oligonucleotides, resulting in a transient knockdown, also called “morphants” [102]. The antisense morpholino, injected into fertilized eggs, targets both maternal and zygotic transcripts; however, off-target effects are notorious, the induced phenotype is transient, and can only be studied for a limited period [102]. With the availability of gene-editing techniques, such as CRISPR/Cas9 technology, a stable KO that allows for the examination of mutant adult zebrafish has become the standard. 

Several tools are available to study glycosidases and glycolipids in mutant zebrafish. Enzyme activity can be evaluated using commercial fluorogenic substrates, such as 4-methylumbelliferone (4MU)-sugars or nitrobenzoxadiazole (NBD)-labeled lipids. Ultrasensitive LC-MS/MS combined with 13C-encoded identical standard techniques are available to quantitatively measure glycosphingolipid abnormalities even in individual zebrafish larvae [103,104]. Cyclophellitol-type activity-based probes (ABPs) allow for the labeling (and visualization) of specific glycosidases [101]. ABPs are particularly useful in zebrafish, as limited zebrafish-specific antibodies are available. In principle, ABPs can be used to visualize the active enzymes in transparent zebrafish larvae.

Zancan et al. created a fish model by targeting the splicing donor site of exon 2 using antisense morpholino oligos and noticed an increase in oxidative stress and Wnt signaling dysfunction [105]. When there was a point mutation in the splicing donor of exon 4 (sa1621), the homozygous mutants displayed a significant decrease in the Wnt signaling pathway. Unsurprisingly, both models developed osteopenia since the canonical Wnt pathway plays an important role in bone homeostasis. This indicated that GCase impairment leads to Wnt signaling pathway dysfunction. 

Another group generated a mutant zebrafish line with a 23 bp deletion in exon 7 (c.1276_1298del) using TALEN (transcription activator-like effector nucleases) genome-editing technology [106]. Sphingolipid accumulation and microglial activation were noticed as early as 5 days post-fertilization (dpf) and it worsened from 8 weeks post-fertilization (wpf). Motor deterioration was seen by 12 wpf. Infiltration of Gaucher cells in the brain and liver was observed along with ubiquitin-positive inclusions in the dopaminergic neurons by 12 wpf [107].

Lelieveld et al. developed *gba1* and *gba2* KO animals as well as a double KO model using CRISPR/Cas9. GlcSph was found to be increased in g*ba1* KO fish and not significantly changed by concomitant g*ba2* deficiency. GlcCer levels were similar between *gba1* KO and WT larvae but increased in *gba2^−/−^* and double KO larvae, likely reflecting a cytosolic GlcCer increase. Upon inhibiting GlcCer synthase (GCS) or overexpressing human *GBA1* in *gba1* KO larvae, levels of GlcCer and GlcSph were reduced.

**Figure 3:**
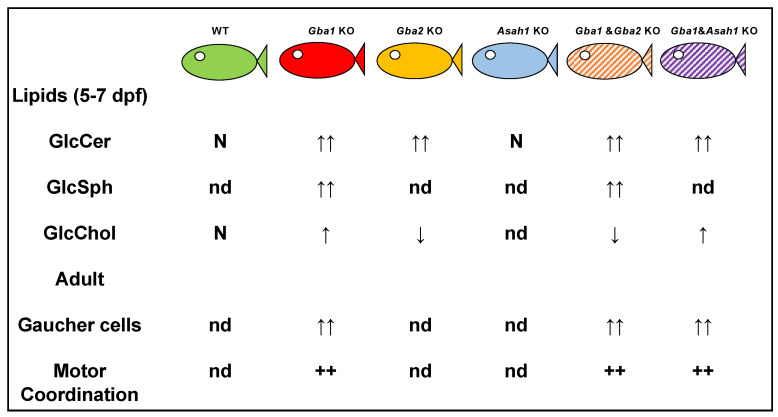
Mutant zebrafish models. Abnormalities noted in glycolipids, presence of Gaucher cells, and abnormal motor coordination (data from Lelieveld et al., 2019 and 2022). g*ba1* encodes lysosomal GCase converting GlcCer to Cer and glucose; *gba2* encodes cytosol-facing GCase converting GlcCer to Cer and generating GlcChol via transglycosylation of cholesterol; *asha1b* encodes acid ceramidase isoform capable of converting GlcCer to GlcSph in lysosomes. N—normal; nd—not determined; ↑—elevated levels; ↑↑—high levels; ↓—decreased levels. ++—severely discordant movement.

In GD, the deficiency of GCase causes lysosomal accumulation of GlcCer, which is partly converted to GlcSph by lysosomal acid ceramidase (aCDase) [108]. Zebrafish have two orthologs for aCDase: Asah1a and Asah1b. Comparison of zebrafish with excessive GlcSph (*gba1* KO fish) or without GlcSph (*gba1*, asah1b double KO fish) strongly indicated that GD fish lacking excessive GlcSph developed an ameliorated course of disease reflected by significantly increased lifespan, delayed locomotor abnormality, and development of an abnormal curved back posture. The loss of tyrosine hydroxylase 1 mRNA, a marker of dopaminergic neurons, was slowed down in the brain of GD fish lacking excessive GlcSph [109]. An illustration of the available zebrafish models is shown in Figure 3.

Recently, Fan et al. expressed a human N370S containing transgene in *gba1*-KO fish and showed that these fish developed resistance towards tuberculosis. Of note, it was also observed that GlcSph at concentrations comparable to those that exist in lysosomes of Gaucher patients’ macrophages potently eliminated Gram-positive bacteria including the tuberculosis bacillus [110].

## 4. Medaka Fish Models of Gaucher Disease

Besides zebrafish, the medaka fish are an increasingly popular research model to unravel human disease conditions. Though zebrafish and medaka fish are both teleosts, they subtly differ in features due to genetic differences. Published are solid comparisons of organ systems of the two types of fish [111,112,113,114]. g*ba1* knockout medaka fish were developed using TILLING (targeting induced local lesions IN genomes) library or TALEN, and survived for approximately 3 months [107,115]. g*ba1* homozygous mutant medaka fish showed accumulation of α-synuclein and loss of dopaminergic and noradrenergic neurons [115]. This mutant demonstrated abnormal rotating swimming movement at 2 months and a bent spine at 3 months. Impairment of the autophagy–lysosome pathway was evident in neurons of *gba1* mutant medaka, suggested by the ubiquitin- and p62-positive aggregates and decreased cathepsin D staining in the lysosome [115]. Nakanishi et al. studied the impact of the deletion of *gba2* in *gba1* KO medaka, which was created using CRISPR/Cas9. It resulted in the exacerbation of GlcCer accumulation with no changes in their lifespan, dopaminergic cell loss, microglial activation, α-synuclein accumulation, or swimming abnormalities. The *gba2* KO medaka did not show any apparent phenotypes, though biochemical analysis revealed alpha-synuclein accumulation in the brain [116].

## 5. Fly Models

In the last decade, a continuously growing number of GD models have been generated in *Drosophila melanogaster*. Considering the many advantages of the fly as a model system, this is not surprising. The *Drosophila* genome comprises four chromosome pairs: chromosome one is the sex chromosome, and the other three are autosomes. Its genes are homologous to more than 75% of genes associated with human diseases [117]. Moreover, many well-studied biological pathways are conserved between human and the fly [118]. Although the anatomy of humans and *Drosophila* differs, they share many genetic, cellular, and physiological properties, as well as similar organs, including the central nervous system, heart, liver, kidney [119], trachea [120], peripheral nervous system [121], and gut [122].

**Figure 4:**
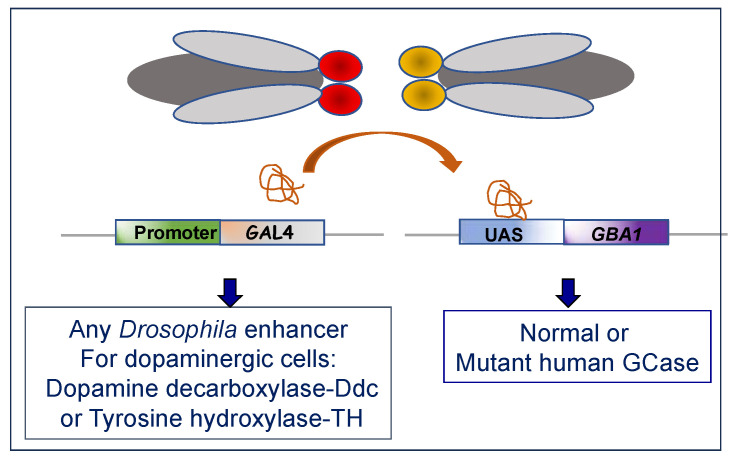
Expression of a foreign gene (transgene) in flies. Through a cross between two flies, a line is established in which the transcription factor GAL4 is expressed from a native promoter coupled to it. GAL4 binds to its promoter, UAS, and activates the expression of a foreign gene coupled to the UAS.

Genetic manipulation of *Drosophila* can generate a wide variety of mutants and transgenes. KO of specific *Drosophila* genes has been achieved by transposon insertions [123] and CRISPR/Cas9-editing technology [124], while RNAi is used to knockdown (KD) genes [125]. While KO or KD of genes allow for the study of loss-of-function models, transgenes are mostly used to study gain-of-function paradigms. For expression of transgenes in the fly, the UAS/GAL4 system is being used (Figure 4). The GAL4 gene is placed under the control of a native gene promoter, while the introduced transgene (usually cDNA) is under the control of the yeast promoter upstream activating sequence (UAS). When co-expressed, GAL4 binds and activates the UAS, which is coupled to the gene of interest. Thus, the expression of a target gene is achieved in the presence of active GAL4 [126,127].

Any manipulated chromosome can be preserved in the population in heterozygosity in combination with a “balancer chromosome”. A balancer chromosome contains chromosomal rearrangements (preventing recombination with its homolog chromosome) [128,129], a dominant marker with a visible phenotype, and a recessive lethal mutation to prevent it from taking over the population [129]. There is a large number of publicly available mutant strains (over 18,000) (http://flypush.imgen.bcm.tmc.edu/pscreen/index. php, accessed on 3 April 2023), each containing a single transposon insertion, generated by the *Drosophila* gene disruption project (GDP) [123]. Adding all the mentioned advantages to the relatively short life cycle of the fly (approximately 90 days at 25 °C, the optimal temperature for flies), which can be manipulated by temperature changes, makes *Drosophila* a powerful genetic tool.

*Drosophila* has two *GBA1* orthologs, *Gba1a* (CG31148) and *Gba1b* (CG31414), which are located on chromosome 3. *Gba1a* is located approximately 2000 bp upstream of *Gba1b*. They are ~2 and ~4 kb in size (3R:23,700,621-23,702,605 and 3R:23704804-23708512), respectively, and are separated by a small non-relevant gene (CG31413), which encodes quiescin sulfhydryl oxidase 4 (Qsox4) (FlyBase.org).

**Figure 5:**
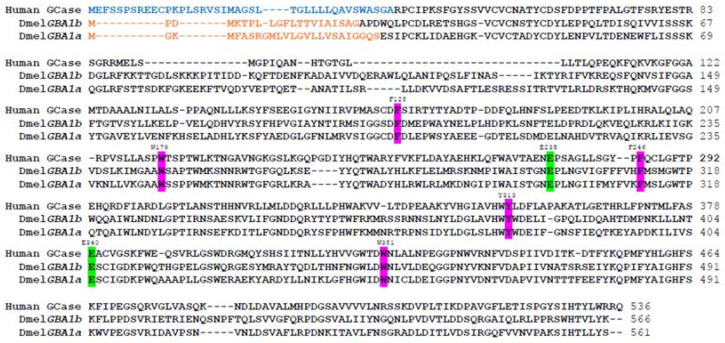
*Drosophila* GBA1 proteins. Multiple sequence alignment analysis comparing human GCase and the two Drosophila GBA1-encoded protein sequences (Dmel *GBA1b*, *GBA1a*). The two amino acids in the active site are highlighted in green. Amino acids that stabilize the active site are highlighted in purple. The known human and predicted fly leader sequences are highlighted in blue and orange, respectively.

The *Gba1a-* and *Gba1b*-encoded proteins share ~50% similarity with human GCase, and more importantly, the two catalytic amino acids, which determine GCase activity, are identical between humans and flies [E340 and E235 in human GCase [2], E259 and E366 in fly *Gba1a-* and *Gba1b*-encoded GCases, respectively] (Figure 5). The same is true for five of the six amino acids that stabilize the substrate in the active pocket of GCase (F128, W179, F246, Y313, W381 in human GCase, F154, W206, F272, Y339, W408, in both fly *GBA1*-encoded proteins, respectively) [130]. 

Interestingly, the *Gba1a*-encoded protein has no GCase-like activity or any other lysosomal enzyme activity [131]. It encodes a protein, which modulates development-regulated apoptosis [131,132]. In contrast, the *Gba1b*-encoded protein has *bona fide* GCase activity [130,133,134]. 

### 5.1. KO Drosophila Models

**Figure 6:**
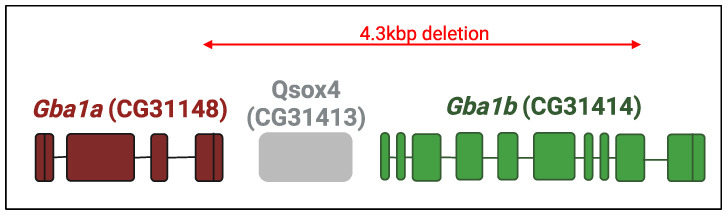
*Drosophila* locus containing the *GBA1* orthologs. Schematic representation of the chromosomal region containing the fly *GBA1* orthologs. *Gba1a* is located 2 Kb upstream of *Gba1b*. CG31413 is a non-relevant gene. Exons of *Gba1a* appear in maroon and those of Gba1b in green. Red arrow represents the deletion created by Davis et al. [134] (see KO *Drosophila* models). Illustration made using BioRender.

To explore the consequences of *Gba1b* deficiency in the fly, Davis et al. created deletions in *GBA1* orthologs using publicly available strains that contain transposable element insertions. Among 200 deletion candidates obtained, there was a 4.3 kb deletion that removed the 33 C-terminal amino acids of the *Gba1a* gene and 433 N-terminal amino acids of the *Gba1b* gene along with CG31413 (see Figure 6). Flies homozygous for the deletion were viable and fertile, with ~40% residual GCase activity compared to that of normal flies. They manifested a shorter lifespan, neural dysfunction, and neurodegeneration along with increased ubiquitin aggregates. Expression of human α-synuclein in the fly (there is no endogenous α-synuclein in the flies), did not enhance the mutant fly phenotypes but there was a mild increase in dopaminergic cell loss [134]. 

Kinghorn et al. established a *Gba1b* KO, and a double KO, for both the *Gba1a* and *Gba1b* genes by ends-out homologous recombination [133]. In the double KO, the open reading frame of *Gba1a* gene was replaced by a *white^hs^* marker gene using homologous recombination, whereas *Gba1b* was disrupted by introducing a stop codon and a frameshift mutation 12 bp downstream to the putative ATG start codon. These flies had a shorter lifespan compared to that of the control flies. Double-mutant flies displayed a lifespan similar to that of single *Gba1b* knockout flies. Both single and double KO flies presented lysosomal defects, progressive age-dependent locomotor deficits, a seven-fold increase in the amount of C16:0 GlcCer in comparison to age-matched controls, autophagic deficits in their brains, synaptic loss and neurodegeneration. Recently, Atilano et al. used this *Gba1b* KO model to study immune and gut pathologies and noticed that these flies had upregulated inflammation, gut dysfunction (increased intestinal transit time, gut barrier permeability, and microbiome dysbiosis), and glial activation in the brain [135].

### 5.2. KI Drosophila Models

**Figure 7:**
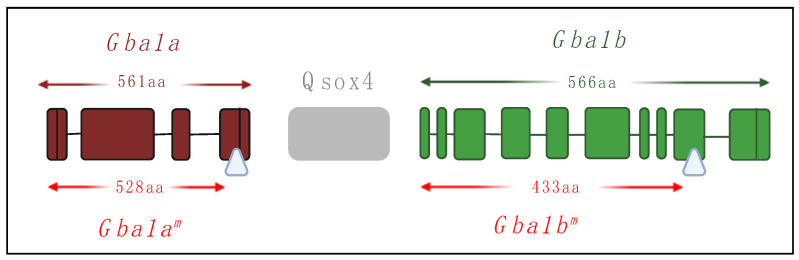
*Drosophila GBA1* orthologs containing the Minos insertions. Schematic representation of the Minos insertion site in each gene. Exons of *Gba1a* appear in maroon and those of *Gba1b* in green. The lengths of *Gba1a-* and *Gba1b*-translated proteins are indicated in maroon and green, respectively. The lengths of the truncated proteins due to Minos insertion (*Gba1a^m^* and *Gba1b^m^*) are indicated in red. The Minos insertion sites are indicated using white triangles. Illustration made using BioRender.

Kawasaki et al. used two available fly lines, each containing a Minos transposable element insertion (MiET) in one of the flies’ *GBA1* orthologs (Figure 7). Homozygous flies, mutated in their *Gba1b* gene, exhibited shorter survival, abnormal climbing, and irregular sleep [136]. 

We also used the above-mentioned Gba1b mutant containing a Minos insertion, which led to translation of a truncated protein containing a 133 C-terminal amino acid deletion (see Figure 7). Flies homozygous for this allele had minimal residual GCase activity and massive substrate accumulation (30-45-fold increase in C14:1 GlcCer levels) with concomitant ER stress, UPR activation, and inflammation [130]. Regarding inflammation in *Drosophila*, two major pathways are associated with immune response activation (and inflammation), the Toll and IMD pathways, which are homologous to the mammalian Toll-like receptor (TLR) and tumor necrosis factor receptor (TNFR) pathways, respectively [137]. Once activated, the receptors lead to signaling pathways that result in the translocation of NF-κB homologous proteins: Dorsal in the Toll pathway and relish in the IMD pathway, from the cytoplasm to the nucleus. These transcription factors initiate the transcription of antimicrobial peptide (AMP) genes in the nucleus. Each pathway is responsible for the transcription of different AMPs. Inflammation could be partly reversed in the *Gba1b* homozygous flies by treatment with ambroxol, a known GCase pharmacological chaperone [138], but there was no effect on the UPR. These results highlight the efficiency of ambroxol as a known anti-inflammatory medication with no effect on UPR since the mutant *Gba1b*-encoded protein lacks 133 C-terminal amino acids, including some which were shown to be important for ambroxol binding [130].

## 6. Other Animal Models of GD

As stated, the present review discusses animal models developed to study the molecular and cellular mechanisms underlying GD. However, it is worth mentioning two naturally occurring models for GD.

### 6.1. Canine Model

No longer available, is an 8-month-old Australian Sydney Silky dog [139] with reduced glucocerebrosidase activity [140], GlcCer accumulation, Gaucher cells, and progressive neurological disease [139].

### 6.2. Ovine Model

A naturally occurring nGD sheep has been identified on a Southdown sheep stud farm in Victoria, Australia [141]. Affected neonatal lambs manifested a severe neurological disease, involving shaking and inability to stand. Their skin at birth is abnormal and thickened. The affected animals survive for only a few days. GCase activity is markedly reduced and GlcCer accumulates (100-fold in the brain and 15-fold in the liver). Lysosomal storage occured in macrophages in the spleen, lymph nodes, and thymus. Pathological changes occured in the central and peripheral nervous system [141]. Mutational analysis found them to be homozygous for the missense mutations c.1142G>A (p.C381Y) and c.1400C>T (p.P467L). The human equivalent [C342Y] to the C381Y mutation leads to an acute neuronopathic phenotype in patients. A more recent study of the nGD sheep confirmed that c.1142G>A in exon 8 of the sheep *GBA1* ortholog is the causative mutation of GD in the Southdown sheep studied [142]. A subsequent investigation of nGD lamb brain focused on lipid abnormalities. Detected were 30- to 130-fold higher GlcCer, and 500- to 2000-fold higher GlcSph concentrations in GD lamb brains compared to wild type. Significant increases in bis(monoacylglycero)phosphate (BMP) and gangliosides [GM1, GM2, GM3] concentrations were also observed [143]. Purified detergent resistant membranes (DRM; lipid rafts) from the occipital cortex and spleen of affected sheep also showed significant increases in the concentrations of GlcCer, hexosylsphingosine, BMP, and gangliosides and decreases in the percentage of cholesterol and phosphatidylcholine [144]. In a very recent study, pathological changes in GD lamb brain were carefully investigated and compared to those in GD patient post-mortem tissue [145]. Significant expansion of the endo/lysosomal system in GD lamb cingulate gyrus was noted. Neurons were found to show shrunken, hypereosinophilic cytoplasm and hyperchromatic or pyknotic nuclei that were also shrunken and deeply Nissl stain positive. Amoeboid microglia were noted throughout the GD brain. Spheroidal inclusions reactive for TOMM20, ubiquitin, and most strikingly, p-Tau were observed in many brain regions in GD lamb brain. Similar abnormalities were noted in GD patient brain, supporting the value of the nGD sheep as a GD research model.

## 7. Conclusions

GD is a rare, autosomal recessive lysosomal disorder. As the first lysosomal and metabolic disease for which enzyme replacement therapy was developed before any animal model was available, it drew a lot of interest and became a model for other lysosomal disorders. Tissue culture has tremendously contributed to the understanding of the molecular and cellular processes underlying this disease, and animal models provide the crucial extra layer to understand the disease at the organismal level and enhance the development of new drugs. 

**Figure 8:**
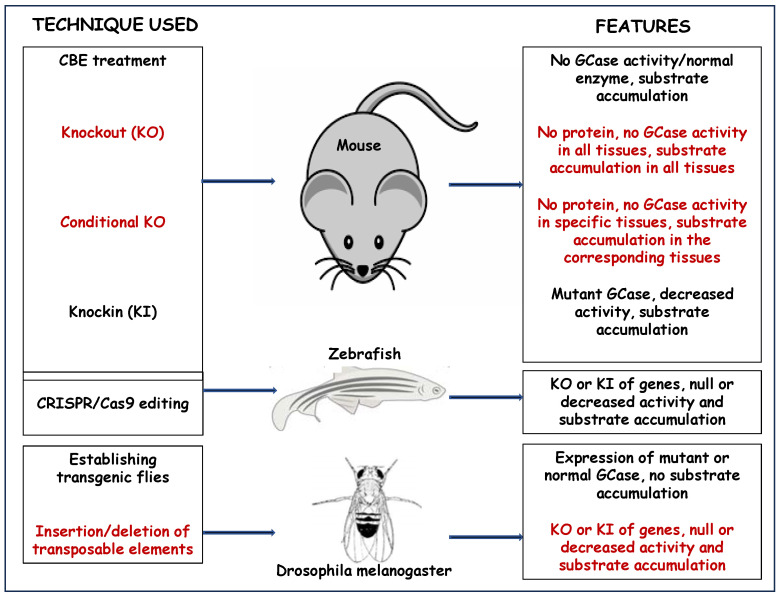
Different animal models used to study mutant *GBA1* variants associated with GD. Shown are key features of animal models used to study GD.

In the present review, we summarized the advantages of the different animal models in studying GD, and Figure 8 is a summary of their characteristics. 

## Data Availability

Not applicable.
